# Hsa-miR-1248 suppressed the proliferation, invasion and migration of colorectal cancer cells via inhibiting PSMD10

**DOI:** 10.1186/s12885-022-10028-1

**Published:** 2022-08-26

**Authors:** Chengxing Wang, Bin Wang, Weijun Liang, Chaorong Zhou, Weixing Lin, Zijie Meng, Wanting Wu, Meimei Wu, Yuehua Liao, Xiaoping Li, Jinglin Zhao, Yaoming He

**Affiliations:** 1 Department of Gastrointestinal Surgery, Jiangmen Central Hospital, Haibang street NO.23, Jiangmen, 529000 Guangdong China; 2 Clinical Experimental Center, Jiangmen Key Laboratory of Clinical Biobanks and Translational Research, Jiangmen Central Hospital, Jiangmen, 529000 Guangdong China; 3 Department of Pathology, Jiangmen Central Hospital, Jiangmen, 529000 Guangdong China; 4 Department of Breast, Jiangmen Central Hospital, Jiangmen, 529000 Guangdong China

**Keywords:** Hsa-miR-1248, Colorectal cancer, PSMD10, Invasion, Migration, Lymph node metastasis

## Abstract

**Background:**

Lymph node metastasis (LNM) is a critical event during the colorectal cancer (CRC) development and is indicative of poor prognosis. Identification of molecular markers of LNM may facilitate better therapeutic decision-making.

**Methods:**

Six pairs of CRC tissues and corresponding adjacent tissues [3 pairs diagnosed as pT1N0M0 (M_Low group) and 3 pairs diagnosed as pT4N2M0 (M_High group)] collected from CRC patients who underwent surgical resection were used. MicroRNA sequencing was performed to screen differential microRNAs involved in CRC LNM. The selected microRNAs were validated in CRC tissues and cell lines using qRT-PCR. The functions of candidate hsa-miR-1248 were evaluated by CCK-8, colony formation, and Transwell assay. The binding of hsa-miR-1248 with its target PSMD10 was confirmed by luciferase activity assay, and the expression of PSMD10 in tissues was detected by droplet digital polymerase chain reaction.

**Results:**

Ninety-five miRNAs were downregulated in carcinoma tissues (M_Low and M_high groups) compared with the normal group. Their expression in M_High group was significantly lower compared with M_Low group. The top 3 were hsa-miR-635, hsa-miR-1248, and hsa-miR-668-3p. After validation in tissues/cell lines, only hsa- hsa-miR-1248 was decreased in high metastatic tissues or SW620 cells compared to low metastatic tissues or SW480 cells. Hsa-miR-1248 was found to inhibit CRC cell viability, proliferation, invasion, and migration. The tumor suppressor effect of has-miR-1248 in CRC cells was attenuated or enhanced by up-regulating or down-regulating PSMD10, respectively.

**Conclusion:**

Hsa-miR-1248 may act as a tumor suppressor gene in CRC by targeting and inhibiting PSMD10, which provides a clue for CRC treatment.

**Supplementary Information:**

The online version contains supplementary material available at 10.1186/s12885-022-10028-1.

## Introduction

Globally, colorectal cancer (CRC) is the 3rd most common cancer and the second leading cause of cancer-related deaths [1]. Lymph node metastasis (LNM) is the most critical event during the progression of CRC and is indicative of poor prognosis. LNM also plays an essential role in the therapeutic decision-making for CRC. Unfortunately, approximately 20% of all CRC patients with no histopathological evidence of LNM (stage I or II disease) develop recurrence within 5 years [2]. Moreover, there is emerging evidence indicating that lymph node micro-metastasis is associated with poor prognosis [3]. Therefore, identification of molecular markers of LNM will help improve therapeutic decision-making in patients with CRC.

MicroRNAs (miRNAs) are short endogenous non-coding RNAs (length 19–21 nucleotides) which regulate their target gene expression post-transcriptionally [4]. MiRNAs are remarkably stable and have been shown to play an essential role in different types of cancer development via competing endogenous RNA (ceRNA) mechanisms. The binding of 5′ nucleotides 2–7 of miRNAs with the 3’UTR of target mRNAs results in the suppression of the expression of target mRNAs [5, 6]. In other words, if miRNAs have been verified to have an abiding site with the predicted mRNAs, the expression of the mRNAs shows a negative correlation with miRNAs. For example, hsa-miR-1248 was reported to bind with cyclin D2 and tumor-promoting gene tripartite motif-containing protein 24 to regulate the progression of non-small cell lung cancer (NSCLC) [7, 8]. Hsa-miR-1248 was also found to regulate the expression of C3A in lung squamous carcinoma cells [9]. However, the role of hsa-miR-1248 in the context of CRC is not well characterized.

Proteasome 26S subunit non-ATPase 10 (PSMD10), also named Gankyrin, has been reported to be overexpressed in CRC and correlated with TNM stage and metastasis [10]. In our recent study, CRC patients with higher PSMD10 expression exhibited a higher risk of occult liver metastases and lower progression-free survival (PFS) rate [11]. However, the relationship between PSMD10 and CRC lymph node metastasis is not clear. Moreover, several miRNAs have been reported to regulate the expression of PSMD10. For example, miR-214 was shown to decrease the proliferation and metastasis of thyroid carcinoma cells through binding with PSMD10 [12]. MiR-1254 was shown to inhibit CRC progression by binding with PSMD10 [13]. However, the relation between hsa-miR-1248 and PSMD10 is unclear.

In this study, we explored the differentially expressed miRNAs among paratumoral normal colon tissues, low lymph node metastatic carcinoma (T1N0M0), and high lymph node metastatic carcinoma (T4N2M0) via miRNA sequencing. Our results indicated that hsa-miR-1248 as a critical miRNA may participate in the CRC LNM. Then, we explored the function of hsa-miR-1248 and its binding site of PSMD10. Hsa-miR-1248 was found to decrease CRC cell proliferation and metastasis by inhibiting PSMD10. This study may provide new insights for CRC therapeutic strategies.

## Materials & methods

### Tissue samples

In this study, we collected 6 pairs of CRC tissues and corresponding paratumoral normal tissues from CRC patients who received surgical resection at the Department of Gastrointestinal Surgery at the Jiangmen Central Hospital, Guangdong Province, China between 2019 and 2020. None of the patients received radiotherapy, chemotherapy or immunotherapy before operation. Patients were diagnosed following the introduction of AJCC 8th Edition: Colorectal Cancer. The samples included three pairs of low metastasis CRC tissues (without lymph node metastasis) diagnosed as pT1N0M0 and their corresponding adjacent tissues, three pairs of high metastasis CRC tissues (with lymph node metastasis) diagnosed as pT4N2M0 and their corresponding adjacent tissues. Informed consent was obtained from all patients. All procedures were approved by the Institutional Research Ethics Committee of the Jiangmen Central Hospital (decision no. JXY202229).

### miRNA sequencing

Low metastasis CRC tissues (M_Low group), high metastasis CRC tissues (M_High group), and their corresponding adjacent tissues were collected for miRNA sequencing, and each group contained three samples. After RNA isolation, RNA from three samples in the same group were mixed, and the RNA from the adjacent normal tissues of the two groups was also mixed as the Normal Control group (Normal group). Then libraries were prepared following the protocol of the TrueSeq Small RNA Sample Prep Kit (Illumina). Subsequently, the libraries were used for sequencing on the Illumina platform. The sequencing data were analyzed using the R package.

### Quantitative real-time polymerase chain reaction (qRT-PCR)

Tissues and cells were collected for RNA isolation using TRIzol (Thermo). Then RNA was reversed into cDNA following the protocol of HiScript® Reverse Transcriptase (Vazyme, Nanjing, China). PCR was performed on the ABI 7900 platform using AceQ® qPCR SYBR Green (Vazyme) as per the manufacturer’s instructions. For calculating the relative miRNA expression, U6 was used as the internal standard; for calculating the relative PSMD10 expression, GAPDH was used as the internal standard. The cycling conditions were as follows: initial denaturation at 95 °C for 10 minutes, then 40 denaturations at 95 °C for 1 minute, and annealing/extension at 56 °C for 1 minute. All samples were made in triplicate and standardized according to the internal control. The fold-changes or relative gene expression levels were calculated based on the 2 − △△Ct method. The primer sequences used in this study are shown in Table 1.

**Table 1:** Primers used in the reactions for real-time RT-PCR

Gene	Sequence (5`– 3`)
GAPDH-up	GAGTCAACGGATTTGGTCGT
GAPDH-dn	GACAAGCTTCCCGTTCTCAG
PSMD10-up	CATGTTACTGGAAGGCGGGG
PSMD10-dn	AGGAGTGTTACCCTCAGTGTCT
hsa-miR-635-RT	GTCGTATCCAGTGCAGGGTCCGAGGTATTCGCACTGGATACGACGGACAT
	ACTTGGGCACTGAAACAAT
hsa-miR-1248-RT	GTCGTATCCAGTGCAGGGTCCGAGGTATTCGCACTGGATACGACTTTAGC
	ACCTTCTTGTATAAGCACTGTGC
hsa-miR-668-3p-RT	GTCGTATCCAGTGCAGGGTCCGAGGTATTCGCACTGGATACGACGTAGTG
	TGTCACTCGGCTCGGCCCA
U6-up	CGCTTCGGCAGCACATATAC
U6-dn	CGAATTTGCGTGTCATCCTTG

### Droplet digital polymerase chain reaction (ddPCR) assay

The PSMD10 expression was also detected by ddPCR. DdPCR was performed as described in a previous study with a few modifications [14]. In brief, RNA was extracted from tissues and reverse-transcribed into cDNA following the protocol of HiScript® Reverse Transcriptase (Vazyme, Nanjing, China), which was used as template. DdPCR was performed on the MicroDrop-100 droplet digital PCR system (Forevergen, China). The primers are shown in Table 1.

### Cell culture

Human low metastasis colon cancer cell line SW480 and Human high metastasis colon cancer cell line SW620 were purchased from the Chinese Academy of Science Cell Bank (Shanghai, China). All cells were cultured in DMEM medium (Gibco, Grand Island, NY, USA), containing 10% FBS at 37 °C in a 5% CO_2_ atmosphere. The cells were passaged at a ratio of 1:4. SW480 and SW620 cell line were used in all cell experiments in this study.

### Plasmid and transfection

The has-miR-1248, miR-1248-sponges, PSMD10, and sh-PSMD10 were PCR-amplified from cDNA and cloned into lentiviral vectors (Yeasen Biotechnology, Shanghai, China). All primers used in the reactions for clone PCR are shown in Table 2. Cell transfection was performed using the Lipofectamine 3000 reagent (Thermo Fisher Scientific, Waltham, MA, USA) as per the manufacturer’s instructions. Briefly, cells were digested, seeded, and cultivated for 24 h before transfection until the attainment of 60–70% confluence. Then, lipofectamine 3000 and relative plasmid were diluted in Opti-MEM individually followed by mixing at 37 °C for 5 min. The medium was replaced with a new medium containing the DNA-lipid mixture. After forty-eight hours, cells were collected for further analysis.

**Table 2:** Primers used in the reactions for clone PCR

Gene	Sequence (5` – 3`)
MiR-1248-up	GAGGATCCCCGGGTACCGGTTTCGAATTCTGGTGAAAAAGG
MiR-1248-dn	CACACATTCCACAGGCTAGCTAACTTCATTCTGTTAGGTCTAATG
MiR-1248-sponges-up	GAGGATCCCCGGGTACCGGTTTTAG
MiR-1248-sponges-dn	CACACATTCCACAGGCTAGCACCTTCT
PSMD10-shRNA-up	CCGGCAGGTTGGTCTCCTCTTCATACTCGAGTATGAAGAGGAGACCAACCTGTTTTTG
PSMD10-shRNA-dn	AATTCAAAAACAGGTTGGTCTCCTCTTCATACTCGAGTATGAAGAGGAGACCAACCTG
PSMD10-CDS-up	ATGGAGGGGTGTGTGTCTAAC
PSMD10-CDS-dn	TTAACCTTCCACCATTCTCTTGAG
PSMD10-WT-up	ACACTTAGCCTGTGATGAGGAGAG
PSMD10-WT-dn	GGGGACAACAACACAACATACAAAG

### Colony formation assay

For colony formation assay, vector-transfected CRC cells were plated in 6-well plates at a density of 500 cells per well and cultured in a humidified atmosphere containing 37 °C for 2 weeks. Cell colonies were washed with phosphate-buffered saline (PBS), fixed with methanol for 30 min, and stained with 0.1% crystal violet (1 mg/mL) for another 30 min. The colony morphologies were captured and manually counted under a light microscope.

### CCK-8 analysis

Cell viability was assessed using Cell Counting Kit-8 (CCK-8) assay. In brief, the indicated CRC cells were seeded on 96-well plates containing complete medium at a density of 1000 cells per well. After cell attachment, the medium was changed, and cells were transfected with relevant vectors, and cultured for 5 days. Cell proliferation was detected every 24 h. Then 10 μL CCK-8 solution was added to each well and the cells were incubated at 37 °C for 4 h. CCK-8 absorbance was measured at 450 nm.

### Invasion and migration assays

Transwell assays were performed to assess the invasion and migration of SW480 and SW620 cells. SW480 and SW620 cells transfected with relevant vectors were first starved for 24 hours. On the next day, the cells were suspended in serum-free L-15 medium, and 4000 cells were seeded in the upper chamber. The lower chamber was filled with 600 μL of L-15 medium containing 20% FBS to induce cell invasion and migration. Following incubation for 48 hours, the membranes with the cells invaded or migrated were fixed with methanol and stained with crystal violet. Then the cells in the membranes were counted under a microscope.

### Luciferase activity assay

miRDB was used to predict the potential binding site in target mRNA PSMD10 of has-miR-1248. The wild-type sequence of PSMD10 (PSMD10-WT) and mutant type sequence of PSMD10 (PSMD10-MUT) were cloned into the pmirGLO vector (Promega) respectively, which was performed by General Biol (Anhui, China). Subsequently, PSMD10-WT or PSMD10-MUT was co-transfected with anti-miR-1248 or anti-miR-NC into SW480 cells. They were also co-transfected with miR-1248 or miR-NC into SW620 cells as well. After transfection for 48 hours, the luciferase activity was measured following the procedure of TransDetect® Double-Luciferase Reporter Assay Kit (Transgen, Beijing, China). PSMD10-Mut can be used as a comparison of the PSMD10-WT, which more intuitively reflects that PSMD10-WT can improve the luciferase activity of SW480 cells and reduce the luciferase activity of SW620 cells.

### Western blot analysis

Cells were lysed by RIPA buffer (Beyond) containing protease inhibitor. Then, protein concentration was evaluated using the BCA method. Protein (25 μg) was separated by 10% SDS-PAGE gel and was transferred to PVDF membrane. Afterward, the membrane was blocked with 5% skim milk and incubated overnight with PSMD10 (ab182576, Abcam), α-tubulin (CST, 3873 s, 1:2000) at 37 °C. Secondary antibody (SA0004–1, Porteintech) was added to the membrane and incubated for 2 h at 25 °C. ECL plus (PE0010, Solarbio) and Tanon-5200 CE (Biotanon, Shanghai, China) were used to observe the protein bands. α-tubulin served as the loading control.

### Statistical analysis

All data were analyzed using Graphpad 8.0 (GraphPad Software Inc., San Diego, CA, USA) and SPSS 10.0 (IBM, Armonk, NY, USA). Continuous variables are presented as mean ± standard deviation and between-group differences were assessed using the Wilcox. test. *P* values < 0.05 were considered indicative of statistical significance.

## Results

### Hsa-miR-1248 showed a relationship with CRC lymph node metastasis

**Fig. 1:**
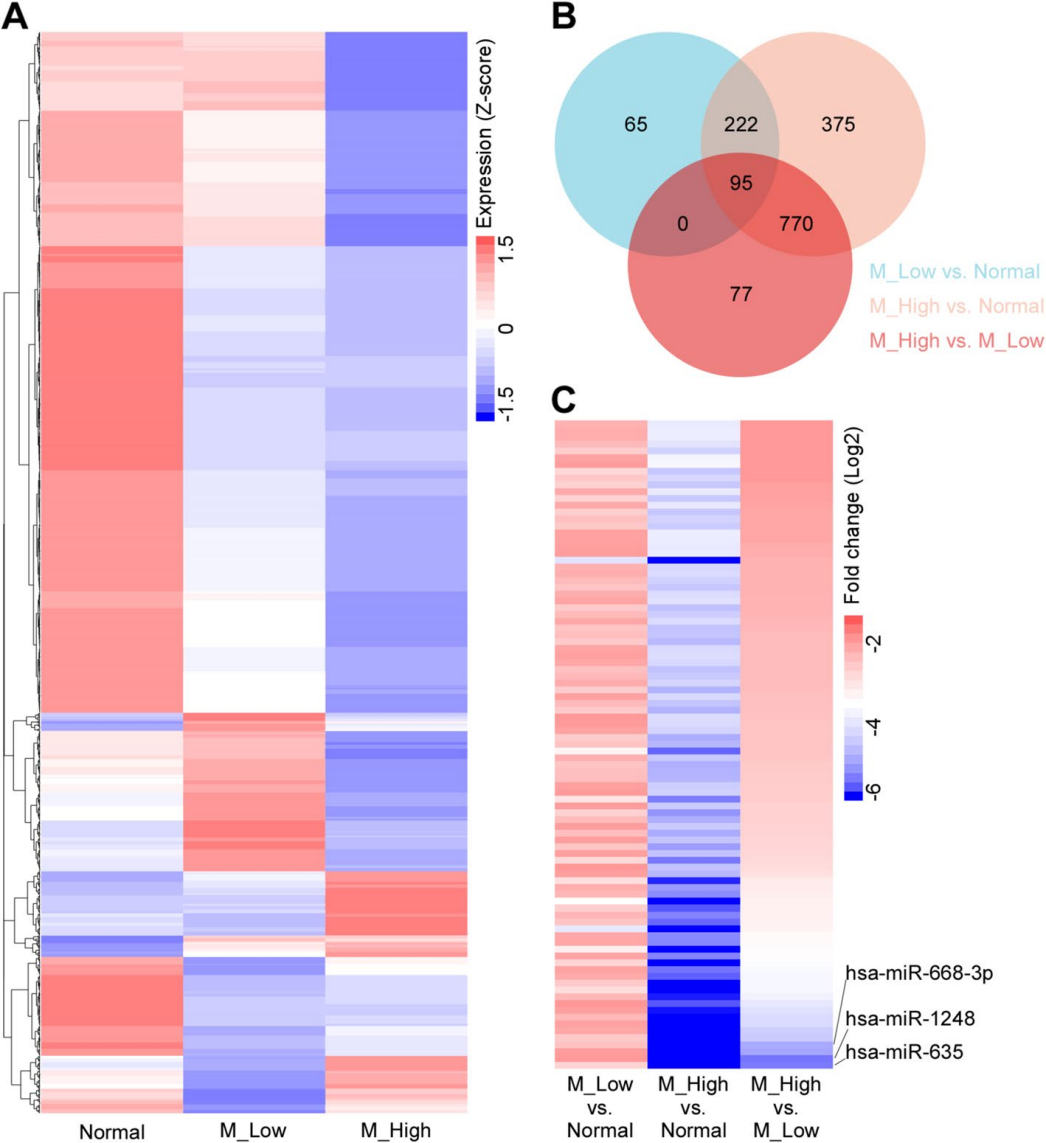
Sequencing results showed a relation of has-miR-1248 with CRC lymph node metastasis. MiRNA sequencing was performed in three groups, i.e., high lymph node metastatic carcinoma tissue group (M_High), low lymph node metastatic carcinoma tissue group (M_Low), and adjacent normal colorectal tissue group (Normal). The expression (*z* score) is shown in the heatmap (Fig. 1A). There are 95 miRNAs down-regulated in carcinoma tissue (M_Low and M_high groups) when compared with the adjacent normal tissue (NC group), and they were further down-regulated in M_High groups when compared with M_Low groups (Fig. 1B). According to the Log2 Fold change in the M_High vs. M_Low, the top 3 differentially expressed miRNAs were hsa-miR-635, hsa-miR-1248, and hsa-miR-668-3p (Fig. 1C). Consequently, we screened out hsa-miR-635, hsa-miR-1248, and hsa-miR-668-3p as the potential miRNAs affecting CRC lymph node metastasis

To explore the miRNA changes between high metastasis (with lymph node metastasis) and low metastasis (without lymph node metastasis) CRC tissues, we performed miRNA sequencing in the three groups, i.e., high metastatic carcinoma tissue (M_High group), low metastatic carcinoma tissue (M_Low group), and adjacent normal colorectal tissue (Normal group). As shown in Fig. 1A, compared to the Normal group, 382 miRNAs were down-regulated in the M_Low group, while 1462 miRNAs were down-regulated in the M_High group. Compared to the M_Low group, 942 miRNAs were downregulated in the M_High group. As shown in the Venn graph (Fig. 1B), 95 miRNAs were down-regulated in carcinoma tissue (M_Low and M_high groups) when compared with the Normal group, and these were also down-regulated in M_High group when compared with the M_Low group. According to the fold-change (log2) in the M_High group compared with the M_Low group, we ranked these 95 miRNAs and harvested the first three, i.e., hsa-miR-635, hsa-miR-1248, and hsa-miR-668-3p (Fig. 1C). According to the miRNA profile, we screened out hsa-miR-635, hsa-miR-1248, and hsa-miR-668-3p as the potential miRNAs affecting CRC LNM.

### Validation of the miRNA expression in CRC tissues and cell lines

**Fig. 2:**
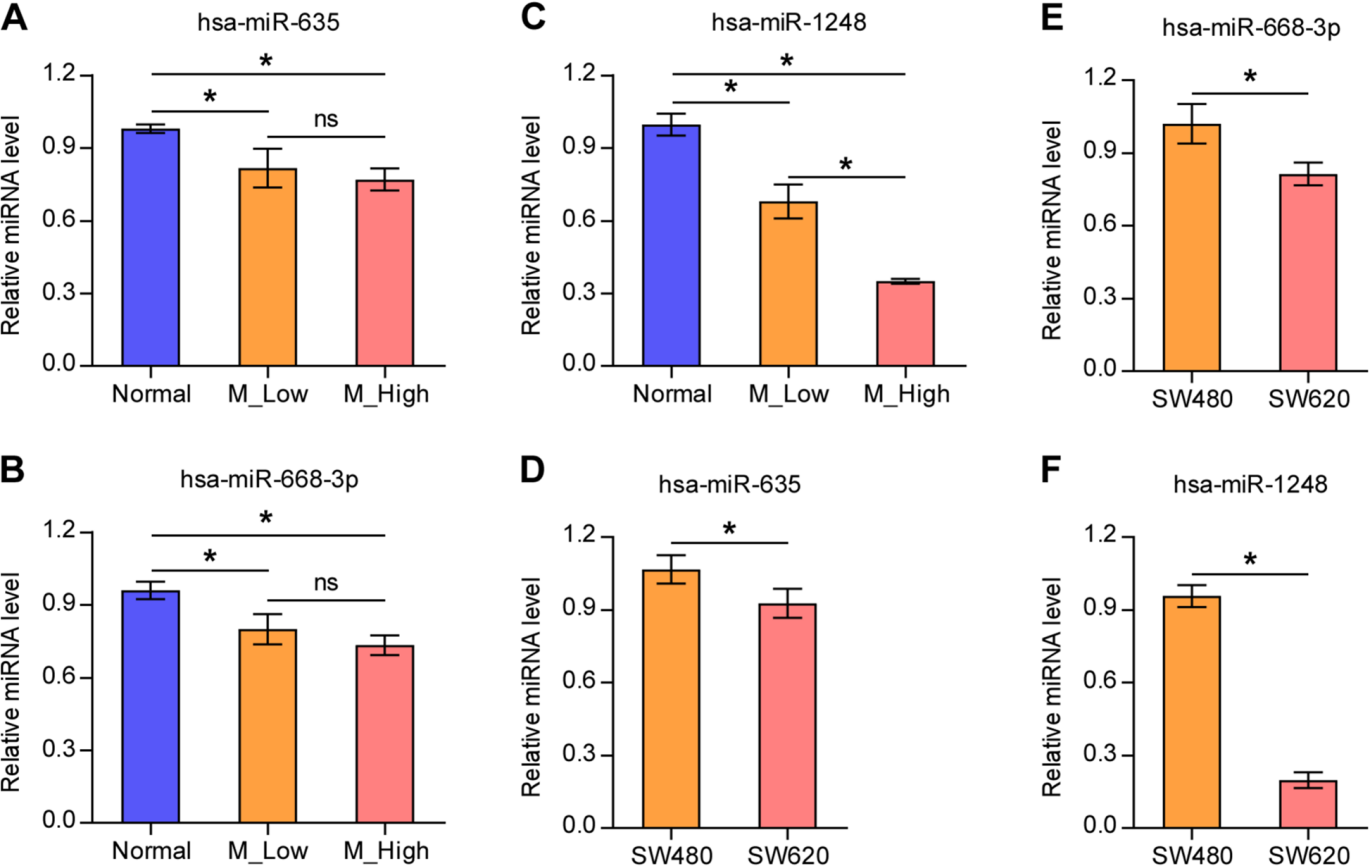
Validation of the expression of miRNAs in CRC tissue and cell lines. **A-C** Results of quantitative real-time polymerase chain reaction showing the expression level of hsa-miR-635, hsa-miR-688-3p, and hsa-miR-1248 in low lymph node metastatic carcinoma tissue, high lymph node metastatic carcinoma tissue, and adjacent normal colorectal tissue. **D-F** Results of quantitative real-time polymerase chain reaction showing the expression level of hsa-miR-635, hsa-miR-688-3p, and hsa-miR-1248 in primary tumor-derived cell line and metastasis-derived CRC cell line. Normal: adjacent normal colorectal tissue; M_Low: low lymph node metastatic carcinoma tissue; M_High: high lymph node metastatic carcinoma tissue. SW480: primary tumor-derived cell line; SW620: metastasis-derived CRC cell line. Mean ± standard deviation values from three independent experiments are presented. *P < 0.05

Then, we performed qRT-PCR assay to verify the expression of these three miRNAs in the sequencing samples. As shown in Fig. 2, hsa-miR-635, hsa-miR-668-3p, and hsa-miR-1248 expression levels in the M_Low and M_High groups were significantly lower than those in the Normal group. These results were consistent with the sequencing data. However, only the hsa-miR-1248 expression level was significantly lower in the M_High group compared with the M_Low group. We also tested the expression of these three miRNAs in human colon cell lines: primary tumor-derived SW480 and metastasis-derived SW620. We found that the expression of hsa-miR-635, hsa-miR-668-3p, and hsa-miR-1248 in SW620 were significantly lower than those in the SW480. Among these, the decrease in hsa-miR-1248 expression was most remarkable. Altogether, only hsa-miR-1248 presented a consistent relationship with CRC LNM, both in CRC tissues and cell lines. Hence, we selected hsa-miR-1248 for further analysis.

### Hsa-miR-1248 inhibits the proliferation, migration, and invasion of CRC cells

**Fig. 3:**
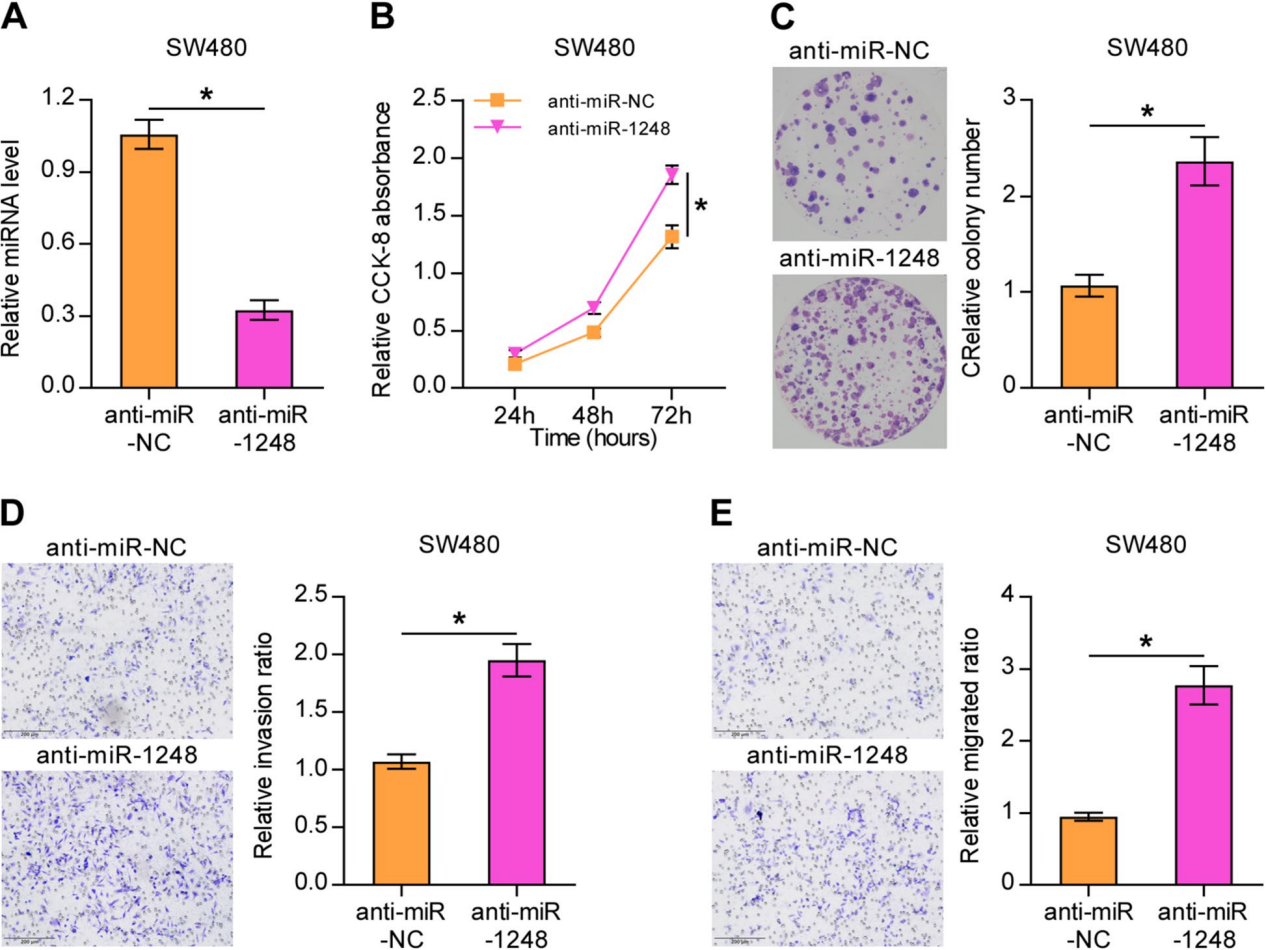
Down-regulation of miR-1248 promoted CRC cell line SW480 proliferation, invasion, and migration. **A** Results of quantitative real-time polymerase chain reaction showing the expression of miR-1248 in SW480 cells transfected with anti-miR-1248 or anti-miR-NC. Results of Cell Counting Kit-8 assay **(B)** and colony formation assay **(C)** showing the proliferation of SW480 cells transfected with anti-miR-1248 or anti-miR-NC. **D, E** Results of Transwell assay showing the invasion and migration of SW480 cells transfected with anti-miR-1248 or anti-miR-NC. Mean ± standard deviation values from three independent experiments are presented. *P < 0.05

As shown in Fig. 3, anti-miR-NC or anti-miR-1248 was transfected into low metastasis colon cell line-SW480, which had a high expression level of miR-1248. The expression of miR-1248 was assessed using qRT-PCR. MiR-1248 expression showed a significant decrease in the anti-miR-1248 group. The CCK-8 assay suggested that SW480 cells transfected with anti-miR-1248 showed much stronger viability at 72 hours. The BrdU cell proliferation assay showed that down-regulation of miR-1248 expression increased the colony formation ability of SW480 cells. In addition, Transwell assays showed that low-expression of miR-1248 had an positive effect on cell invasion and migration.

**Fig. 4:**
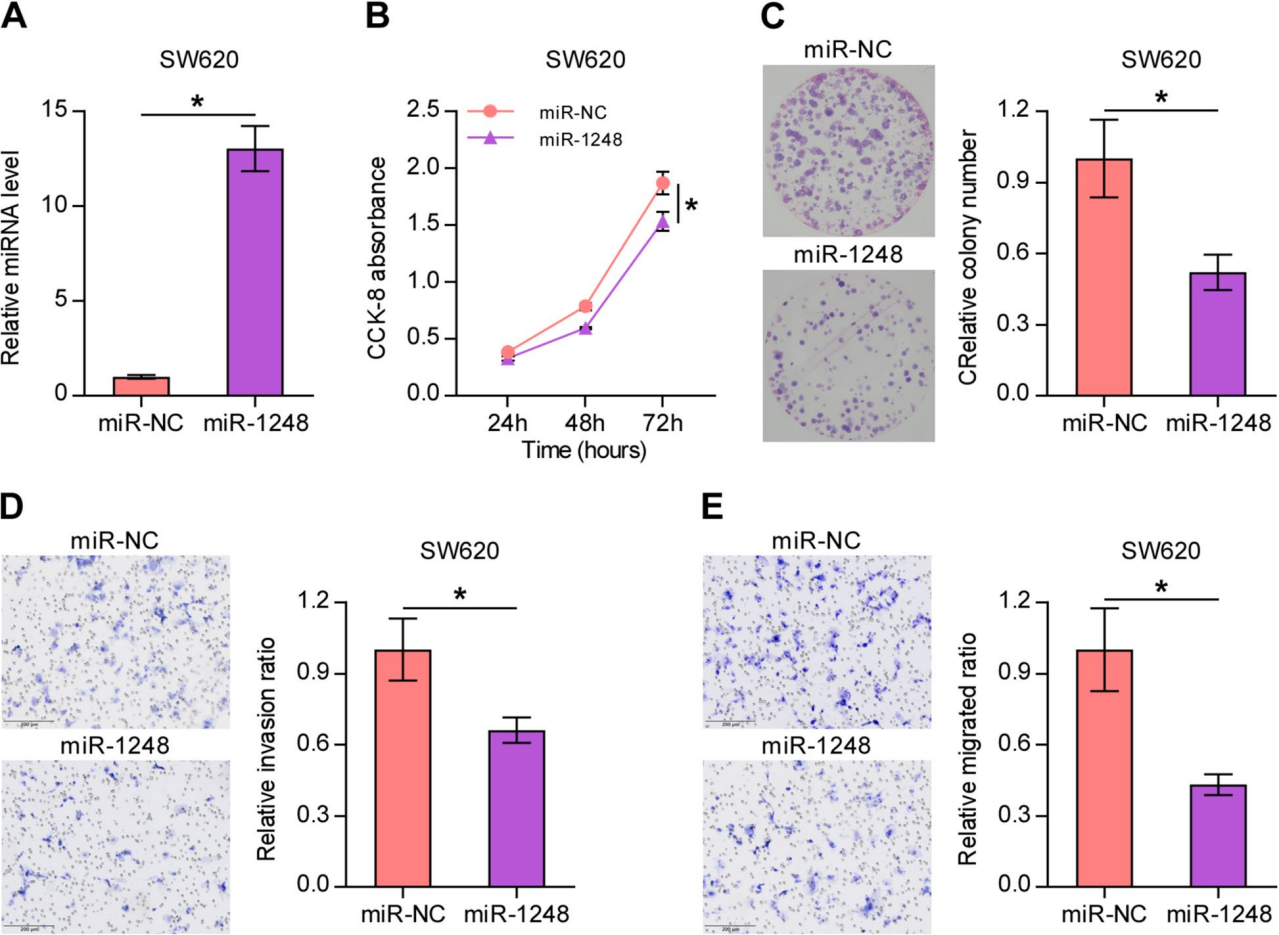
Overexpression of miR-1248 inhibited the proliferation, invasion, and migration of CRC cell line SW620. **A** Results of quantitative real-time polymerase chain reaction showing the expression of miR-1248 in SW620 cells transfected with miR-1248 or miR-NC. **B** Results of Cell Counting Kit-8 assay and **(C)** colony formation assay showing the proliferation of SW620 cells transfected with miR-1248 or miR-NC. **D, E** Results of Transwell assay showing the invasion and migration of SW620 cells transfected with miR-1248 or -miR-NC. Mean ± standard deviation values from three independent experiments are presented. *P < 0.05

Moreover, as shown in Fig. 4, miR-NC mimic and miR-1248 mimic were also transfected into high metastasis colon cell line-SW620, which had a low expression level of miR-1248. The qRT-PCR results showed a significantly higher miR-1248 expression in the miR-1248 mimic group SW620 cell line. The CCK-8 assay, BrdU cell proliferation assay, and Transwell assay were also performed to evaluate the cell viability, proliferation, invasion, and migration. The results indicated that upregulation of hsa-miR-1248 decreased the viability and proliferation of SW620 cells, and inhibited their migration and invasion. These data suggest that hsa-miR-1248 inhibited the proliferation, migration, and invasion of CRC cells, in turn affecting CRC progression and metastasis.

### Hsa-miR-1248 direct binding with PSMD10 and regulate its expression

**Fig. 5:**
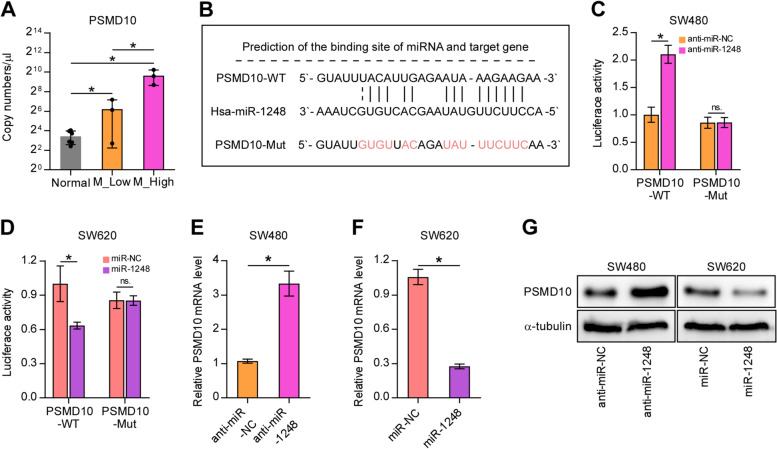
hsa-miR-1248 directly binds with *PSMD10* and regulates its expression in CRC cells. **A** Results of Droplet digital polymerase chain reaction (ddPCR) assay showing the mRNA expression of PSMD10 in the Normal, M_low, and M_High tissues; each group contained three samples. **B** Sequences of Hsa-miR-1248, PSMD10-WT, and PSMD10-Mut; **(C)** Results of dual-luciferase assay showing the relative luciferase activity in SW480 cells transfected either with PSMD10-WT or PSMD10-Mut. **D** Results of dual-luciferase assay showing the relative luciferase activity in SW620 cells transfected either with PSMD10-WT or PSMD10-Mut; **(E)** Results of quantitative real-time polymerase chain reaction showing the expression of *PSMD10* in SW480 cells transfected with anti-miR-1248 or anti-miR-NC. **F** Expression of *PSMD10* in SW620 cells transfected with miR-1248 or miR-NC; **(G)** Western blots showing the expression of PSMD10; α-tubulin served as the loading control (The edges of the blots are cropping, and the full-length blots are presented in Supplementary Fig. 1). The results in **(C-F)** are presented as mean ± standard deviation of three independent experiments. *P < 0.05

We next investigated the relationship between *PSMD10* expression levels and miR1248 (Fig. 5). We also performed ddPCR to measure the expression of *PSMD10* in the CRC tissues and their corresponding adjacent tissues collected for sequencing. We found that PSMD10 expressed a high copy number in CRC tumor tissues compared with the adjacent normal tissue, and the copy number was further elevated in M_High tissue compared with M_Low tissue, which showed an inverse correlation with miR-1248. We further verified the regulatory relationship between miR-1248 and *PSMD10*. The binding site was predicted by miRDB, then confirmed by luciferase activity assay. Anti-miR-1248 co-transfected with PSMD10-WT increased the luciferase activity of SW480 cells compared with that co-transfected with PSMD10-Mut. Moreover, miR-1248 co-transfected with PSMD10-WT decreased the luciferase activity of SW620 cells compared with that co-transfected with PSMD10-Mut. These results revealed that hsa-miR-1248 showed direct binding with *PSMD10* and regulated its expression.

### Has-miR-1248 influences the viability, migration, and invasion of CRC cells by regulating PSMD10

**Fig. 6:**
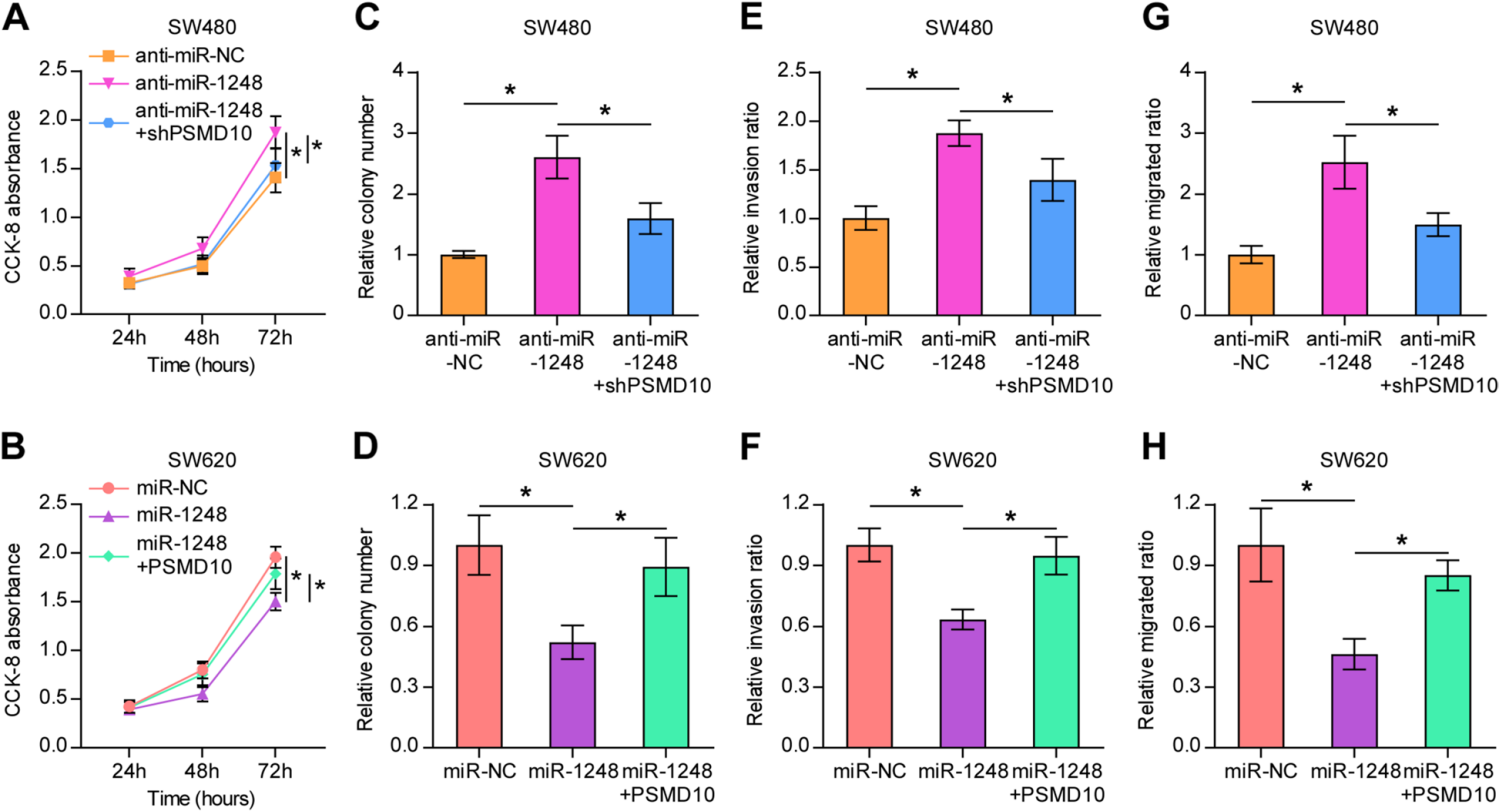
miR-1248 regulated the *PSMD10* expression to influence the proliferation, invasion, and migration of CRC cells. Cell proliferation, invasion, and migration of SW480 cells in the anti-miR-NC, anti-miR-1248, and anti-miR1248 + shPSMD10 were assessed by CCK-8 assay (**A**), colony formation assay (**C**), and Transwell assay (**E**, **G**). Cell proliferation, invasion, or migration of SW620 cells transfected with miR-NC, miR-1248, or miR1248 + PSMD10 were assessed using CCK-8 assay (**B**), colony formation assay (**D**), and Transwell assays (**F**, **H**). Mean ± standard deviation values from three independent experiments are presented. *P < 0.05

In previous experiments, hsa-miR-1248 had been confirmed to inhibit the viability, proliferation, migration, and invasion of CRC cells. As PSMD10 was verified as the target of hsa-miR-1248, the expression of PSMD10 was suppressed in the hsa-miR-1248 mimics group and was enhanced in the anti-hsa-miR-1248 group. Then we performed CCK-8 assay, BrdU cell proliferation assay, and Transwell assay in CRC cell lines transfected with various vectors. As shown in Fig. 6, suppression of miR-1248 significantly enhanced the viability, proliferation, invasion, and migration of SW480 cells, but these effects were attenuated when the SW480 cells were co-transfected with shPSMD10. In addition, we also performed the same experiments in SW620 cells. Upregulation of miR-1248 significantly weakened the viability, proliferation, invasion, and migration of SW620 cells. However, these effects were attenuated when SW620 cells were co-transfected with PSMD10-mimic, which was consistent with the results obtained above. To summarize, hsa-miR-1248 was considered to regulate the proliferation, migration, and invasion of CRC cells partly through PSMD10.

## Discussion

Lymph node metastasis (LNM) is the most common path of metastasis of CRC. LNM is associated with a poor prognosis and is a key determinant of the therapeutic strategy. However, the traditional pathological diagnosis of LNM may not satisfy the requirement of individualized treatment. Therefore, identification of potential molecular biomarkers that can accurately predict LNM and provide a reference for advanced intervention is a key research imperative. MiRNAs play an important role in the development of cancer; moreover, some studies have indicated the predictive value of miRNAs for LNM in CRC [15, 16]. In the present study, we adopted a sample mixture strategy prior to sequencing, which attenuated the variability between individual samples. The relationship between the candidate micro-mRNA and CRC LNM was further validated in tumor tissues.

After sequencing, we found that 95 miRNAs were down-regulated not only in the carcinoma tissue compared with the adjacent normal tissue, but also further decreased in the high lymph node metastasis CRC tissue compared with low lymph node metastasis one. The top 3 miRNAs were hsa-miR-668-3p, hsa-miR-635, and hsa-miR-1248. Their relationship with tumorigenesis has previously been reported. For example, miR-635 was shown to be a potential inhibiting factor in NSCLC and gastric cancer [17, 18]. MiR-668-3p expression was shown to be significantly increased in hepatocellular carcinoma [19]. Hsa-miR-1248 was found to be downregulated in NSCLC and lung squamous carcinoma cells [7–9]. However, there is a paucity of studies about the role of miR-635, miR-668-3p, and hsa-miR-1248 in CRC. Our tissue and cell line validation experiments found that these three miRNAs were downregulated in CRC tumor tissues and exhibited a lower expression in the high metastasis CRC cell line SW620.

Interestingly, in addition to the significant downregulation in SW620 cells, only the expression of hsa-miR-1248 was further decreased in the high lymph node metastasis CRC tissue. Furthermore, we also confirmed that hsa-miR-1248 was associated with cell viability, proliferation, migration, and invasion. Therefore, the molecular mechanism of hsa-miR-1248 was worth further elucidation.

As miRNAs often regulate mRNA expression to promote or inhibit cancer, their roles in carcinogenesis and tumor progression vary in different types of tumors. For instance, hsa-miR-1248 was shown to be upregulated in gastric cancer [20] and esophageal squamous cancer tissue [21] and to act as an oncogene. However, its expression level was decreased in NSCLC tissue and it acted as a tumor suppressor gene [7, 8]. The differential expression of critical genes in these tumors contributes to this divergence. Hence, the key target mRNAs of hsa-miR-1248 in CRC were further predicted. *PSMD10* was earlier shown to be a potential target for cancer therapy [22]. It was also found to act as an oncogene in many types of cancer, such as hepatocellular carcinoma [23], clear cell renal cell carcinoma [24], papillary thyroid carcinoma [25], testicular cancer [26], and osteosarcoma [27]. *PSMD10* was earlier reported to be overexpressed in CRC and its expression level correlated with TNM stage and metastasis [10]. In our recent research, *PSMD10* was shown to be a promising biomarker of occult liver metastasis in CRC patients [11]. Furthermore, in the present research, we also found a potential association of *PSMD10* with LNM. This was also consistent with the relationship between hsa-miR-1248 and CRC LNM. Then, the luciferase activity assay confirmed the binding of hsa-miR-1248 to PSMD10, and the CRC cell line transfected with hsa-miR-1248 relative vector resulted in the regulation of *PSMD10*. Collectively, these findings indicated that *PSMD10* was a target of hsa-miR-1248. Moreover, the antitumor effect of hsa-miR-1248 was suppressed or enhanced by up-regulating or down-regulating the expression of *PSMD10*. These results implied that mi-R1248 plays a tumor suppressor role in CRC partially via *PSMD10*.

Some limitations of our study need to be acknowledged. Only a small number of clinical samples were used in this study. Future studies should confirm the expression of hsa-miR-1248 in CRC using a larger number of clinical samples. Secondly, we did not assess the correlation between hsa-miR-1248 and clinical features of CRC. Moreover, in vivo studies are required to assess the function of the hsa-miR-1248/PSMD10 axis in animal models.

## Conclusion

We observed decreased expression of hsa-miR-1248 in CRC tissues with lymph metastasis. The hsa-miR-1248 was found to bind with *PSMD10* and inhibit its expression. PSMD10 was overexpressed in CRC and showed a positive correlation with LNM. Therefore, hsa-miR-1248 overexpression can inhibit the proliferation and motility of CRC cells by inhibiting the expression of *PSMD10*. Collectively, this study identified the potential involvement of a new axis hsa-miR-1248/PSMD10 in the development of CRC. Our findings may provide new insights for CRC therapy.

## Supplementary Information

**Additional file 1.**

**Additional file 2.**

**Additional file 3.**

**Additional file 4.**

## Data Availability

All data generated or analyzed during this study are included in this published article [and its supplementary information files].

## Abbreviations

LNM: Lymph node metastasis
CRC: Colorectal cancer
miRNAs: MicroRNAs
ceRNA: Competing endogenous RNA
NSCLC: Non-small cell lung cancer
PSMD10: Proteasome 26S subunit non-ATPase 10
TNM: Tumor node metastasis
miRDB: miRNA target prediction and functional annotations online database
