# Supplementary figures and images for: Hsa-miR-1248 suppressed the proliferation, invasion and migration of colorectal cancer cells via inhibiting PSMD10
Source: BMC Cancer. 2022 Aug 26;22:922. doi: 10.1186/s12885-022-10028-1 (PMC9414407; doi:10.1186/s12885-022-10028-1)

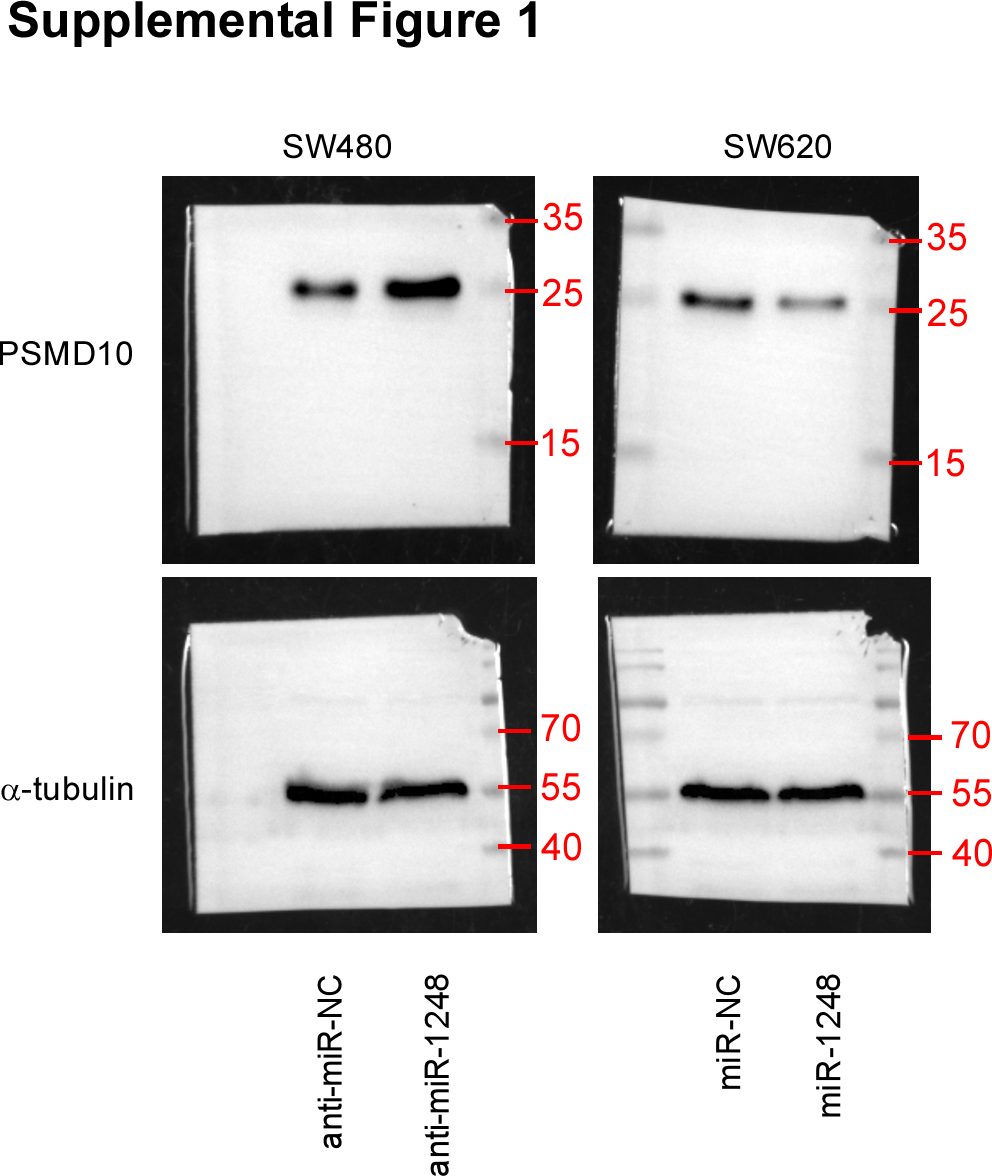

Supplement: Supplementary file 1 — Additional file 1. [file 12885_2022_10028_MOESM1_ESM.tif]

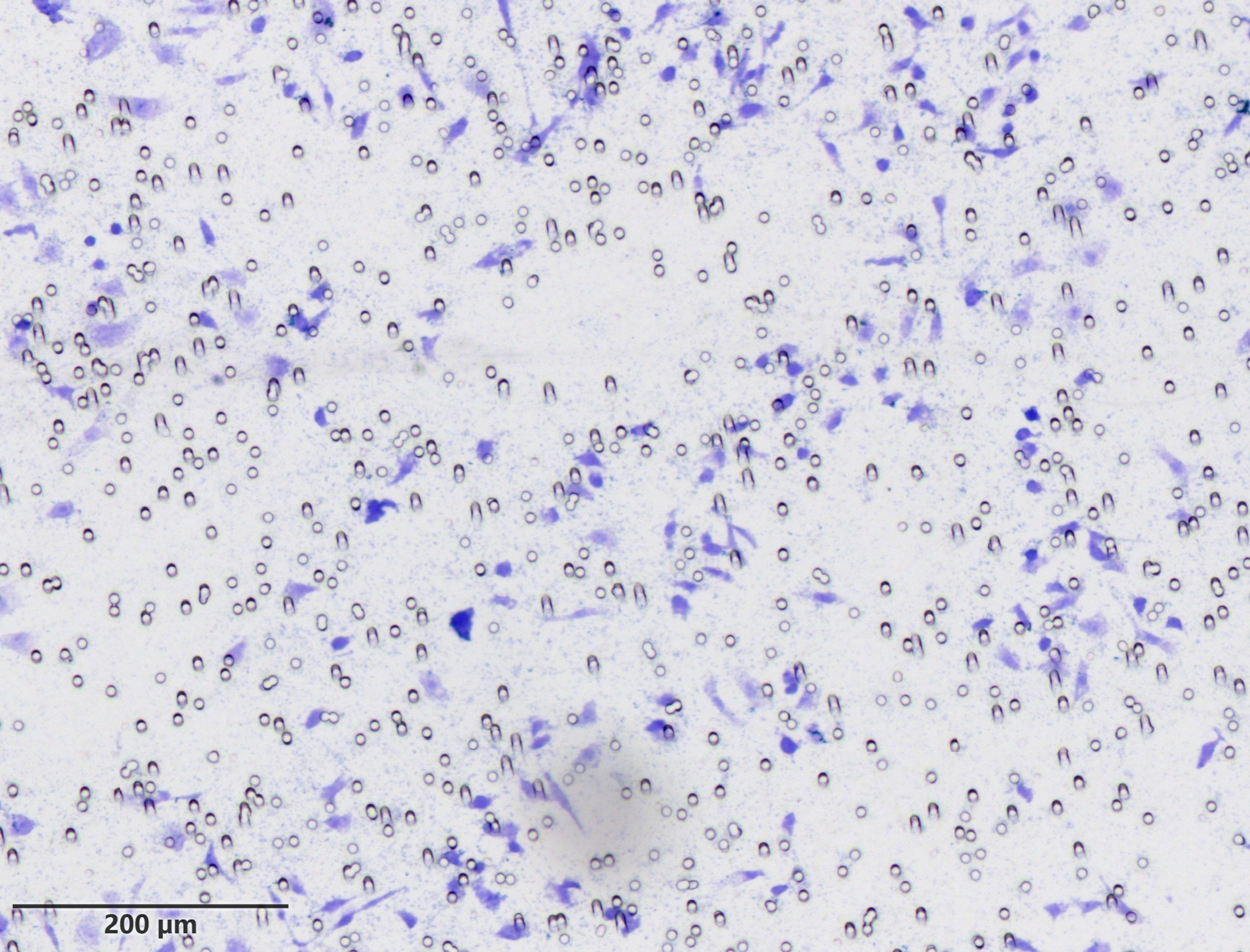

Supplement: Supplementary file 2 — Additional file 2. [file 12885_2022_10028_MOESM2_ESM.zip › cell experimental data/antiNC-SW480-invasion.jpg]

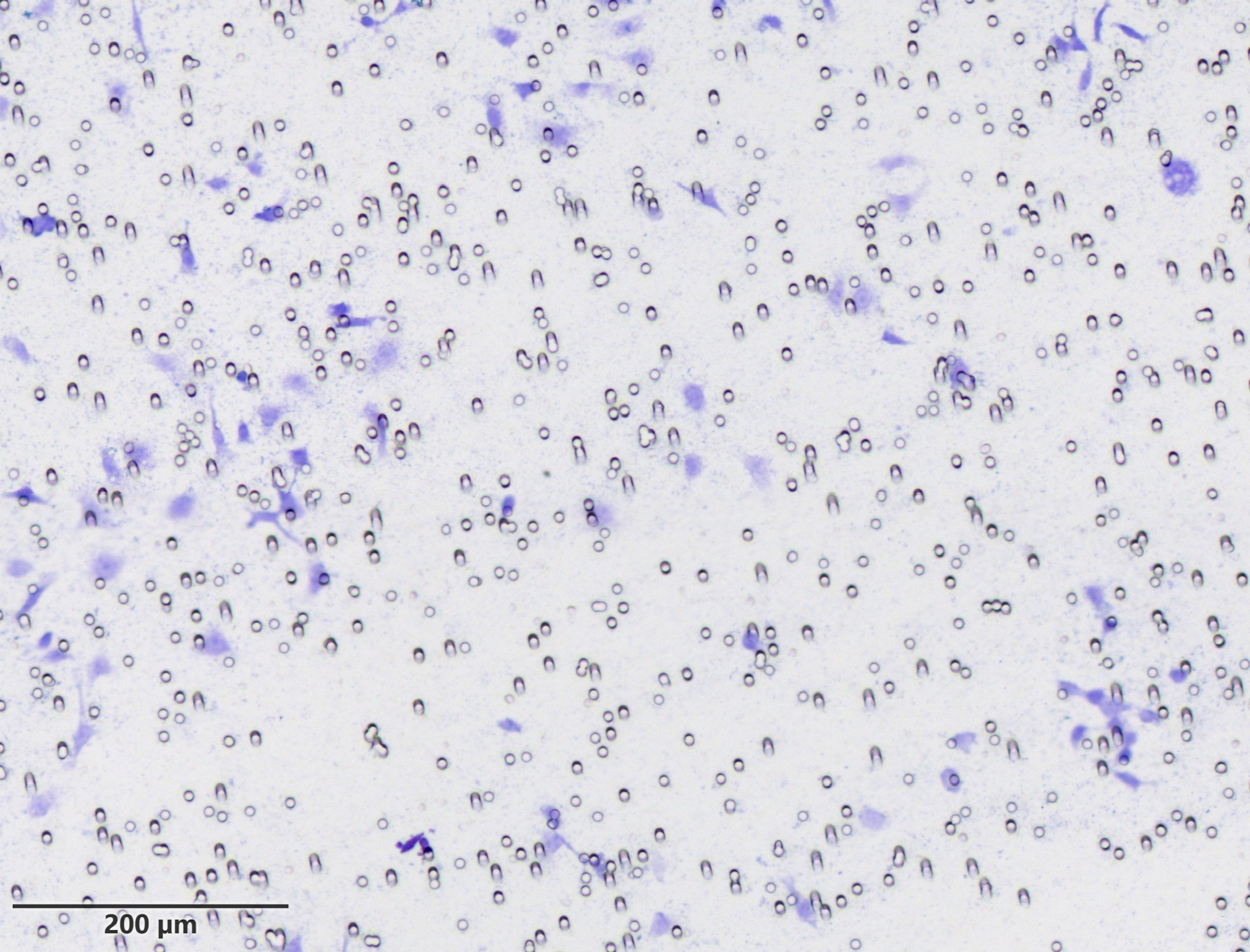

Supplement: Supplementary file 2 — Additional file 2. [file 12885_2022_10028_MOESM2_ESM.zip › cell experimental data/antiNC-SW480-migration.jpg]

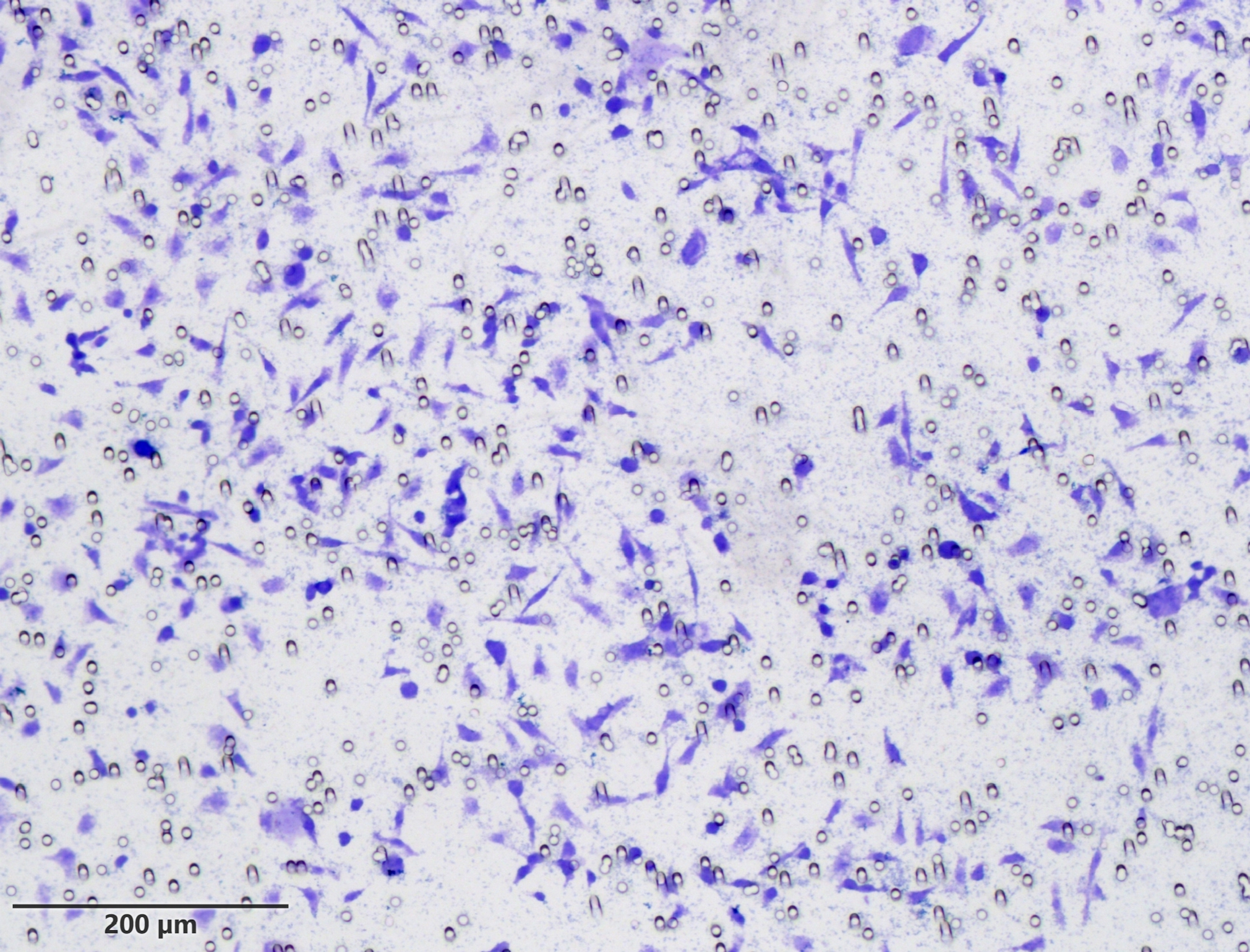

Supplement: Supplementary file 2 — Additional file 2. [file 12885_2022_10028_MOESM2_ESM.zip › cell experimental data/antimiR1248-SW480-invasion.jpg]

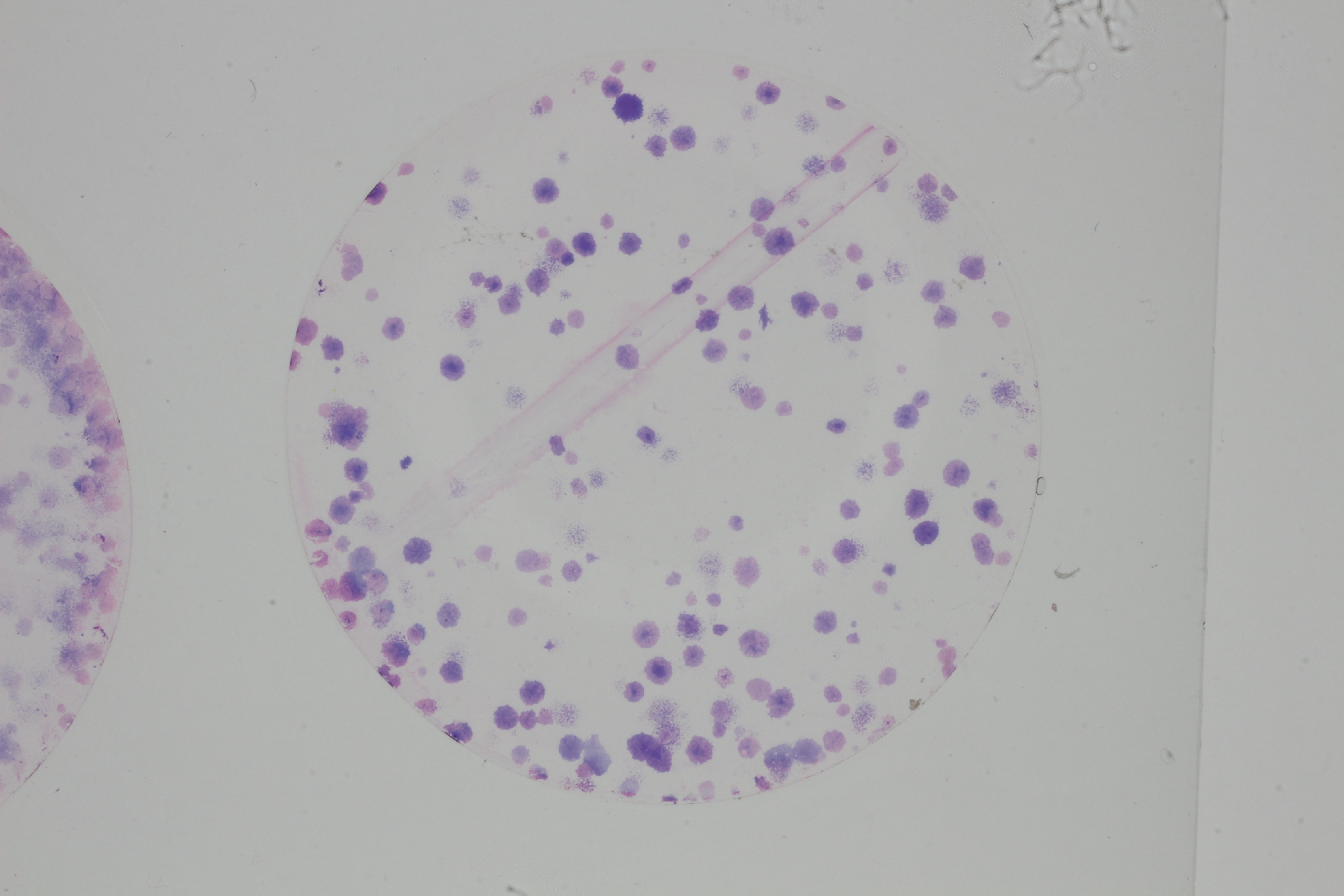

Supplement: Supplementary file 2 — Additional file 2. [file 12885_2022_10028_MOESM2_ESM.zip › cell experimental data/miR-1248.jpg]

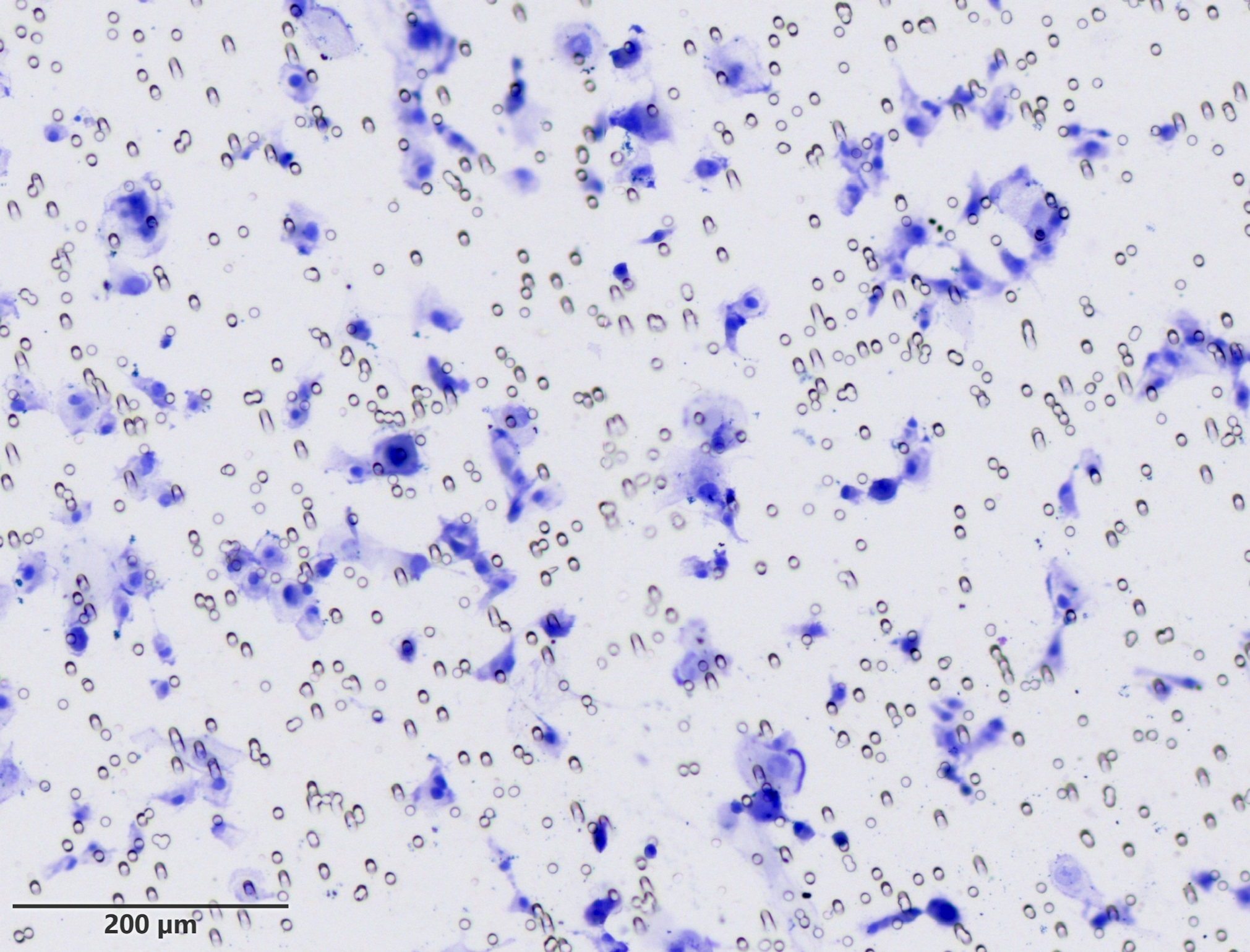

Supplement: Supplementary file 2 — Additional file 2. [file 12885_2022_10028_MOESM2_ESM.zip › cell experimental data/NC-SW620-migration.jpg]

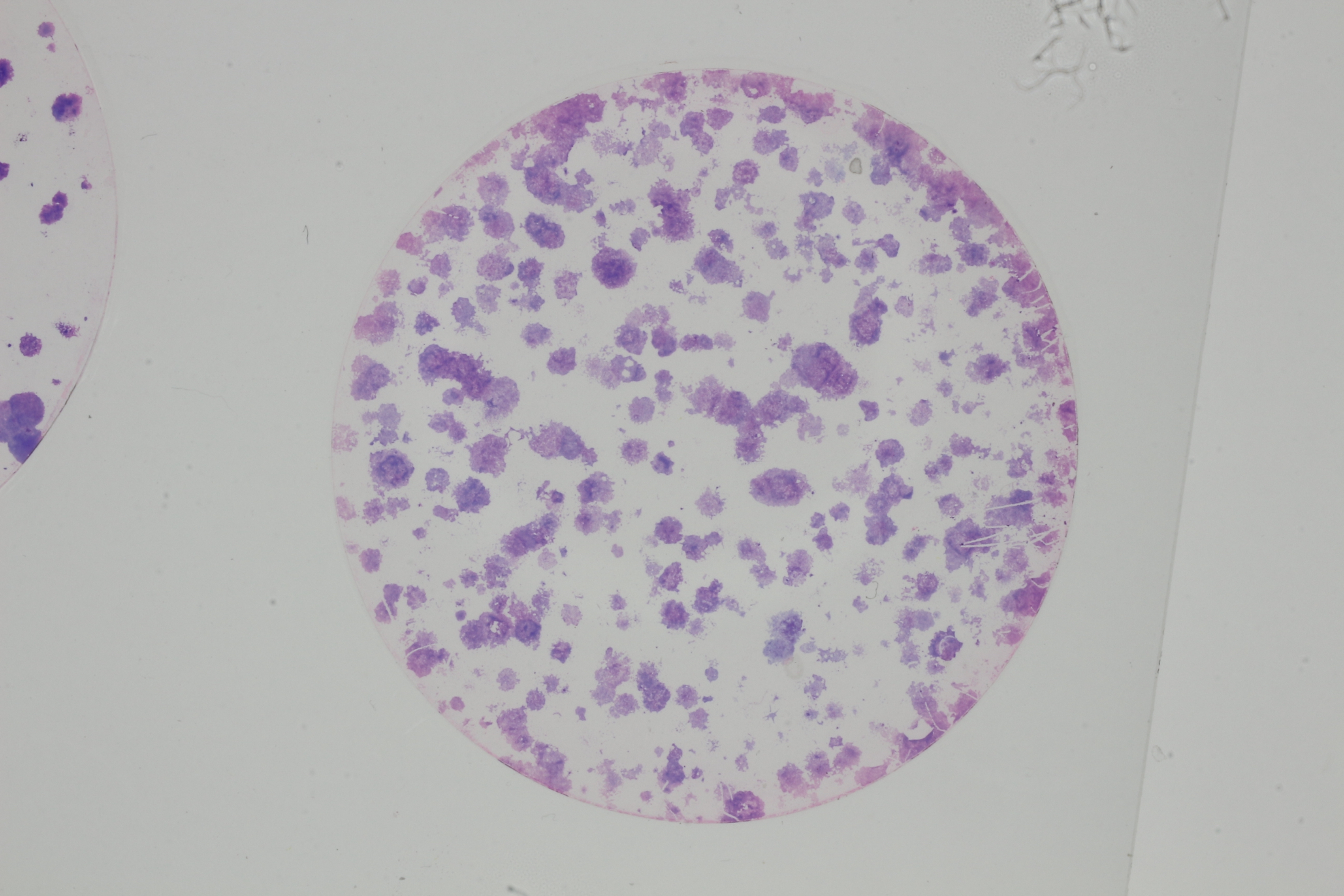

Supplement: Supplementary file 2 — Additional file 2. [file 12885_2022_10028_MOESM2_ESM.zip › cell experimental data/miR-NC.jpg]

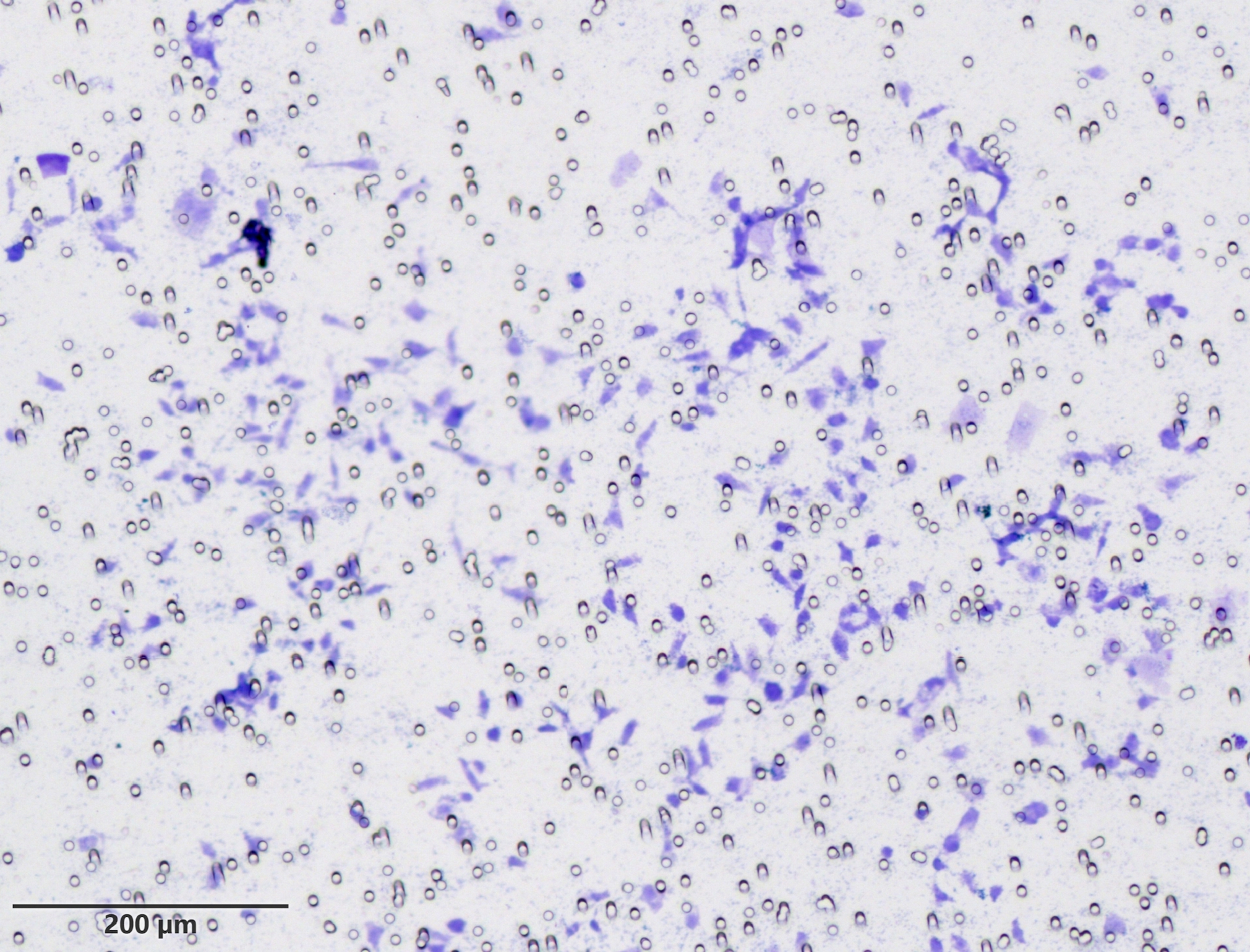

Supplement: Supplementary file 2 — Additional file 2. [file 12885_2022_10028_MOESM2_ESM.zip › cell experimental data/antimiR1248-SW480-migration.jpg]

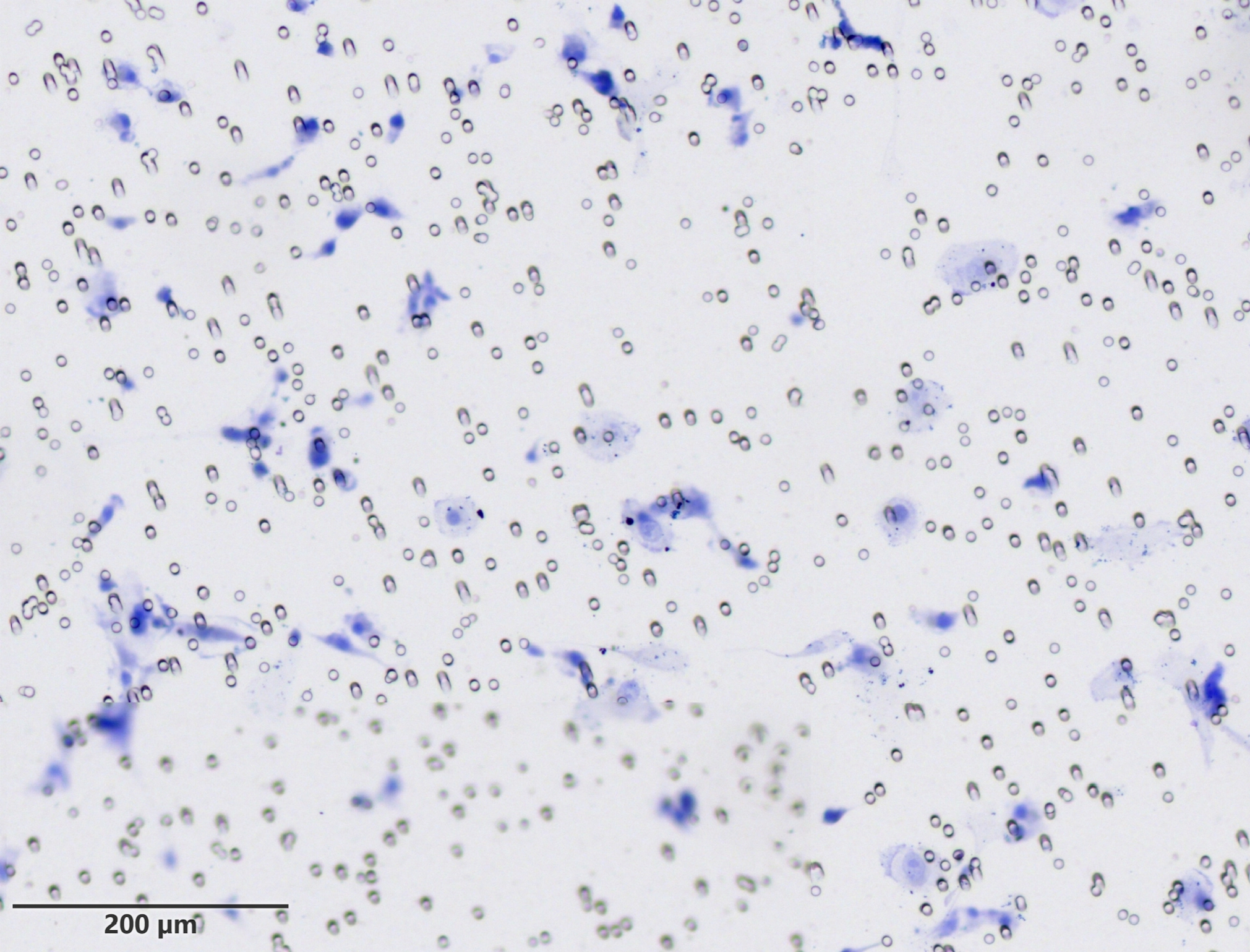

Supplement: Supplementary file 2 — Additional file 2. [file 12885_2022_10028_MOESM2_ESM.zip › cell experimental data/miR1248-SW620-invasion.jpg]

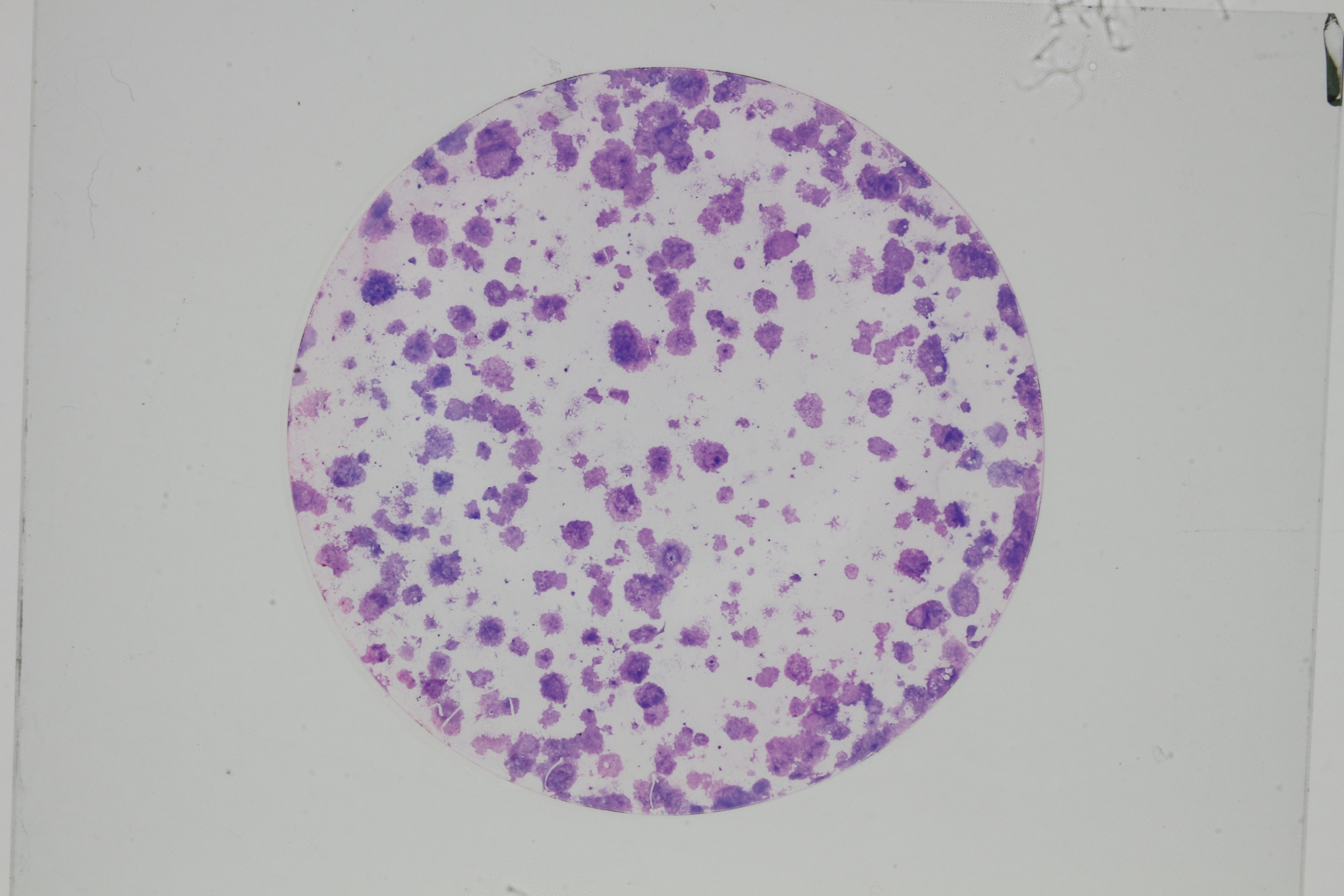

Supplement: Supplementary file 2 — Additional file 2. [file 12885_2022_10028_MOESM2_ESM.zip › cell experimental data/anti-miR-1248.jpg]

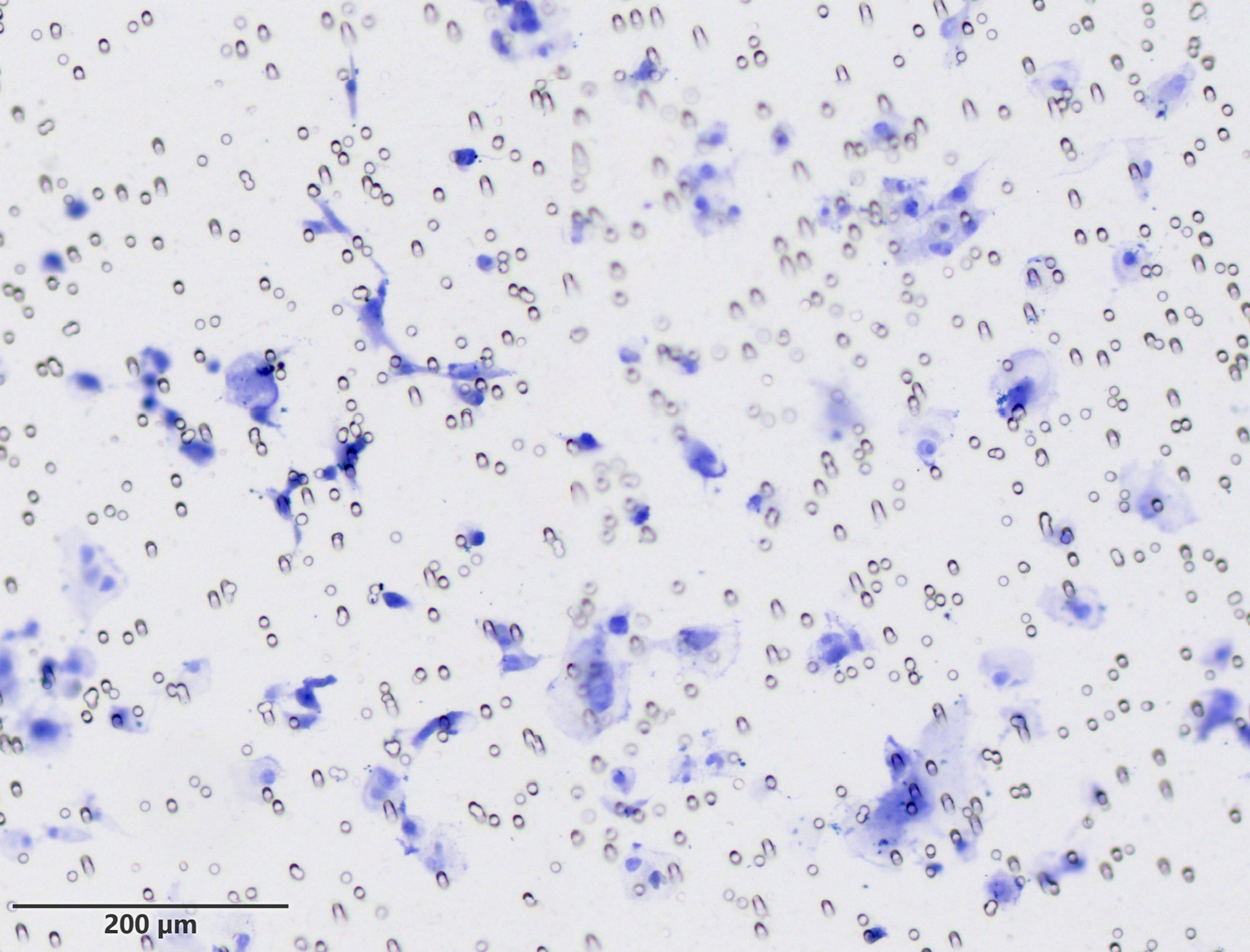

Supplement: Supplementary file 2 — Additional file 2. [file 12885_2022_10028_MOESM2_ESM.zip › cell experimental data/miR1248-SW620-migration.jpg]

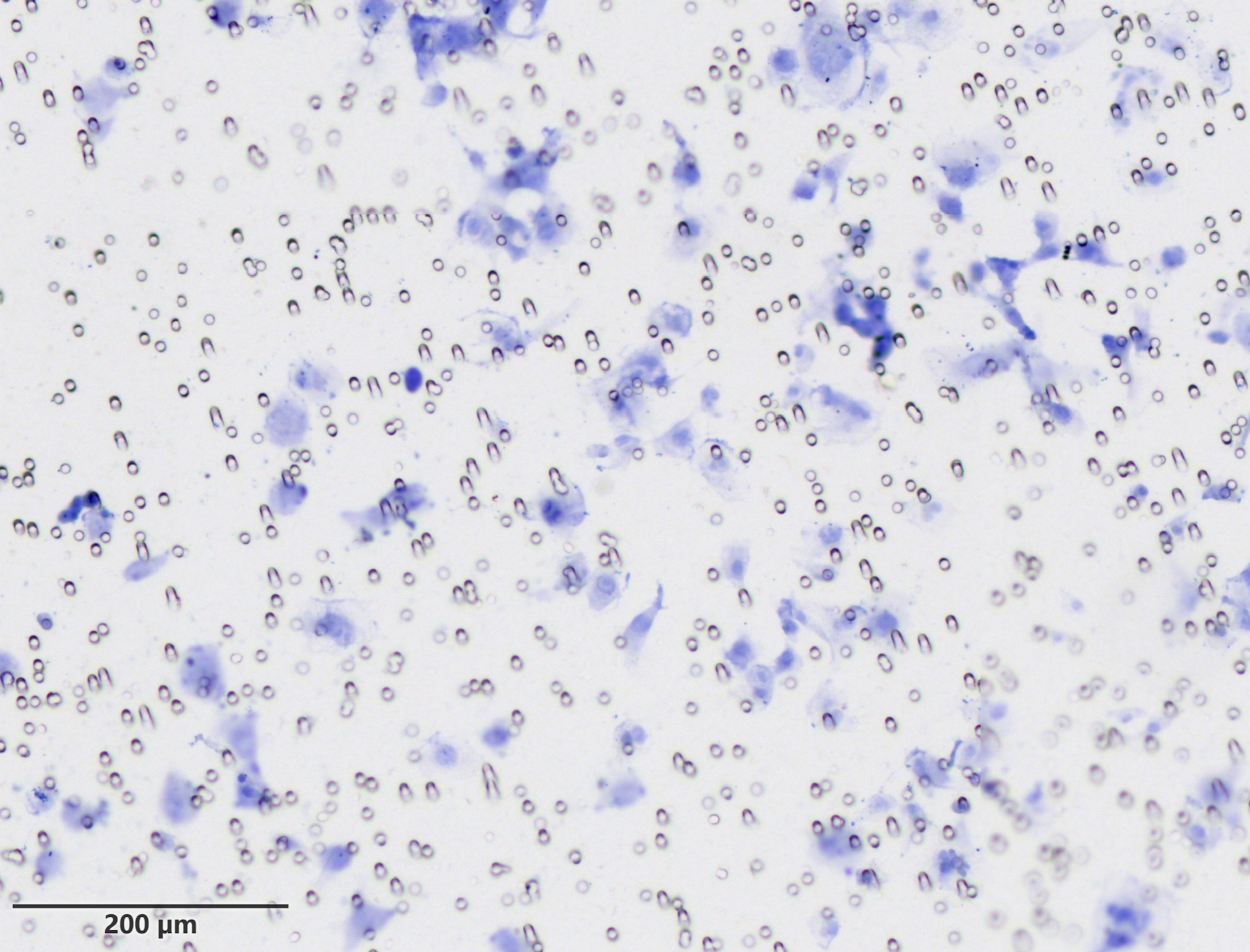

Supplement: Supplementary file 2 — Additional file 2. [file 12885_2022_10028_MOESM2_ESM.zip › cell experimental data/NC-SW620-invasion.jpg]

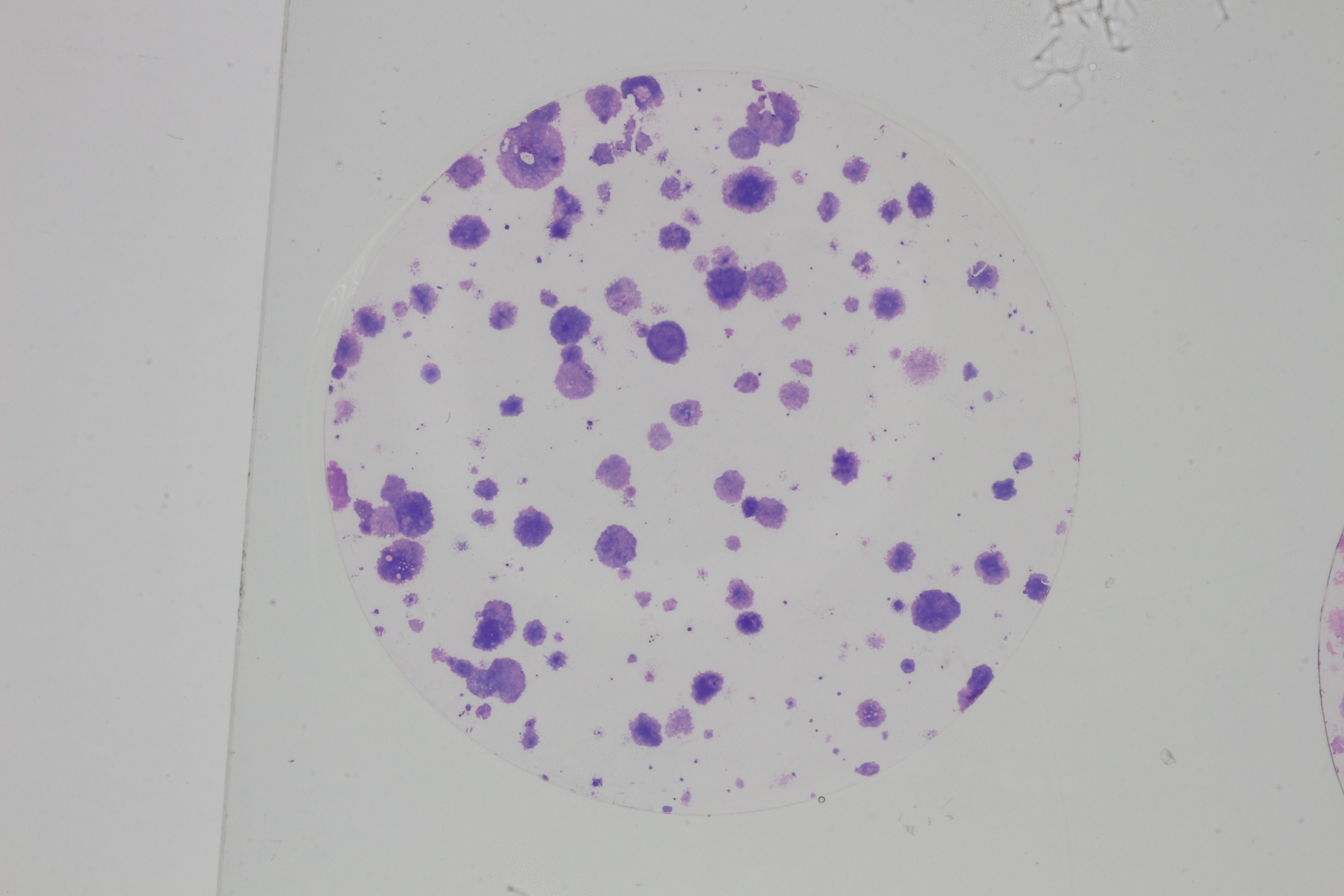

Supplement: Supplementary file 2 — Additional file 2. [file 12885_2022_10028_MOESM2_ESM.zip › cell experimental data/anti-miR-NC.jpg]
